# Supplementary material for: A fungal plant pathogen discovered in the Devonian Rhynie Chert
Source: Nat Commun. 2023 Dec 1;14:7932. doi: 10.1038/s41467-023-43276-1 (PMC10692235; doi:10.1038/s41467-023-43276-1)
Supplement: Supplementary file 3 — Description of Additional Supplementary Files [file 41467_2023_43276_MOESM3_ESM.pdf]

Description of Additional Supplementary Files

File Name: Supplementary Movie 1
Description: Movie of figure 2B. Confocal laser scanning microscopy, false-coloured for z-stack depth; thin section NHMUK-V16430.

File Name: Supplementary Movie 2
Description: Movie of figure 2D. Confocal laser scanning microscopy; thin section NHMUK- V16430.
